# Digital Twins for Targeted Therapy in Head and Neck Cancer: From Molecular Stratification to Resistance-Aware Combination Strategies

**DOI:** 10.3390/curroncol33090566

**Published:** 2026-09-17

**Authors:** Francisca Gonçalves, Marta Gonçalves, Pedro Barata, Nuno Vale

**Affiliations:** 1 PerMed Research Group, RISE-Health, Faculty of Medicine, University of Porto, Alameda Professor Hernâni Monteiro, 4200-319 Porto, Portugal; fsgoncalves@med.up.pt (F.G.); mmcgoncalves@med.up.pt (M.G.); 2 Laboratory of Personalised Medicine, Department of Community Medicine, Health Information and Decision (MEDCIDS), Faculty of Medicine, University of Porto, Rua Doutor Plácido da Costa, 4200-450 Porto, Portugal; 3 Faculty of Health Sciences, University of Fernando Pessoa, Rua Carlos da Maia, 296, 4200-150 Porto, Portugal; pbarata@ufp.edu.pt; 4 Unidade Local de Saúde de Santo António, E.P.E., Largo Professor Abel Salazar, 4099-001 Porto, Portugal; 5 RISE-Health, Faculty of Health Sciences, University of Fernando Pessoa, Praça 9 de Abril, 349, 4249-004 Porto, Portugal; 6 RISE-Health, Department of Community Medicine, Health Information and Decision (MEDCIDS), Faculty of Medicine, University of Porto, Rua Doutor Plácido da Costa, 4200-450 Porto, Portugal

**Keywords:** head and neck squamous cell carcinoma (HNSCC), targeted therapy, digital twin, precision oncology, resistance mechanisms, combination therapy, multi-omics

## Abstract

Head and neck squamous cell carcinoma (HNSCC) remains clinically challenging because of its marked inter- and intra-tumour heterogeneity, the dynamic emergence of therapeutic resistance, and the limited ability of current biomarkers to guide treatment adaptation over time. Recent advances in digital twin (DT) technology have motivated the development of patient-specific, continuously updated computational models capable of integrating multi-scale data to support precision oncology. However, no current DT framework for HNSCC combines molecular stratification, longitudinal monitoring of resistance, pharmacodynamic modelling, and clinical decision support within a single adaptive system. In this review, we critically examine the current state of DT-enabled approaches for targeted therapy in HNSCC and propose a conceptual framework for their future clinical implementation. We discuss how genomic and multi-omic stratification, mechanistic imaging models, pharmacokinetic/pharmacodynamic modelling, longitudinal liquid biopsy (ctDNA and exosomes), ex vivo functional testing, toxicity prediction, and artificial intelligence could be integrated into a continuously updated patient-specific model. We further examine the biological mechanisms driving resistance to targeted therapies and immunotherapy, highlighting how these dynamic processes should inform adaptive therapeutic decision-making. We discuss existing DT-like approaches and evaluated them according to their capacity to fulfil the DT criteria, while also presenting DT-enabling technologies. Among the frameworks currently available, the deep reinforcement learning-based DITTO platform represents the closest approximation to a clinically relevant HNSCC digital twin. However, it does not yet incorporate molecular signalling networks, longitudinal resistance biomarkers, or multimodal biological data. We therefore identify the integration of these complementary data layers as the principal challenge and opportunity for the next generation of DTs. Collectively, this review provides a conceptual DT framework in which four main data domains could fulfil different roles within the DT. By sharing different parameters across these layers, the framework could forecast emerging resistance and update model predictions longitudinally. Such a DT could support biomarker-guided patient stratification, adaptive treatment selection, rational combination therapies, toxicity prediction, and future clinical trial design in HNSCC. We also highlight challenges and limitations that need to be addressed for future clinical translation, including data integration, interpretability, clinical validation, workflow integration, and ethical and regulatory considerations.

## Simple Summary

Head and neck squamous cell carcinoma (HNSCC) is difficult to treat because tumours can differ greatly from one patient to another and can quickly adapt to targeted medicines, leading to treatment resistance. “Digital twin” (DT) approaches aim to create a patient-specific, continuously updated virtual model by combining information from routine clinical data, tumour molecular testing, medical imaging, blood-based signals, and laboratory models grown from a patient’s tumour. This review examines how we could integrate different sources of information into a future DT framework for HNSCC. Such a framework could potentially help clinicians select the most appropriate targeted treatment for each person, anticipate when resistance is emerging, and design safer and more effective drug combinations, including immunotherapy and radiotherapy. To date, no DT for HNSCC integrates these different data layers. In this review, we describe the future framework and also discuss what is needed for these tools to be trusted and used in practice, such as clear validation, data standardisation, and integration into hospital workflows. Ultimately, DTs could support more adaptive and personalised care and improve the design of future clinical trials in head and neck oncology.

## 1. Introduction

Head and neck cancer (HNC) comprises a group of malignant neoplasms arising from the mucosal epithelium of the upper aerodigestive tract, including the oral cavity, pharynx and larynx. As the seventh most common cancer worldwide, it accounted for 792,280 new cases and 424,066 deaths in 2021 [1]. Approximately 90% of these malignancies are histologically classified as head and neck squamous cell carcinomas (HNSCC) [2].

The development of HNC is associated with well-established risk factors, including tobacco use, alcohol consumption, infection with oncogenic viruses, such as Epstein-Barr virus (EBV) and human papillomavirus (HPV), betel nut chewing, certain sexual practices (e.g., oral sex), chronic mechanical irritation, and occupational exposures [1,3]. Statistically, there has been an increase in the number of cases linked to HPV infection, in contrast to other risk behaviours such as smoking and drinking [1]. A review from 2025 revealed that around 60 to 70% of newly diagnosed cases of oropharyngeal cancer in the US and Europe were caused by HPV infections [2].

Treatment strategies in HNSCC are typically determined by tumour histology, anatomical site, and stage at diagnosis, and often require a multidisciplinary approach [3]. For disease characterised by local invasion and regional lymph node involvement, treatment commonly includes surgical resection combined with radiotherapy and, in selected cases, chemotherapy. In advanced-stage disease, systemic therapies, particularly immunotherapy, either alone or in combination with chemotherapy are considered [2,3,4]. However, these treatment strategies do not fully account for the marked biological heterogeneity of HNSCC. As a result, there has been a growing shift away from focusing on uniform therapeutic approaches toward precision oncology, with emphasis on targeted therapies and biomarker-driven patient stratification. These strategies aim to tailor treatment to specific molecular subgroups, thereby improving clinical outcomes and reducing unnecessary toxicity [5]. Nevertheless, their clinical implementation remains challenging due to a number of limitations, which include tumour heterogeneity [6,7], and development of resistance to treatment [5]. In fact, head and neck cancer exhibits substantial interindividual variability in its molecular and clinical characteristics. Marked differences are observed between HPV-positive (e.g., frequent alterations in PIK3CA) and HPV-negative tumours (e.g., loss of TP53 and CDKN2A), which consequently differ in terms of aetiology, biological behaviour, prognosis, and response to therapy [6]. Moreover, intratumoural heterogeneity plays a critical role in shaping therapeutic outcomes. The immune landscape of HNSCC is highly heterogeneous, reflecting significant variation in immune cell composition within individual tumours, which contributes to inconsistent responses to both targeted therapies and immunotherapies [6]. Genetic heterogeneity within tumours can be quantitatively assessed using metrics such as mutant-allele frequencies. Notably, elevated mutant-allele tumour heterogeneity (MATH) scores are observed in a substantial proportion of HNC cases and have been associated with significantly worse survival outcomes, independent of established clinical factors, thereby underscoring the clinical relevance of tumour heterogeneity [7].

The development of therapeutic resistance represents a major barrier to effective treatment in HNSCC. Targeted therapies, for example, the only anti-EGFR approved for HNSCC, cetuximab, show limited efficacy partly due to mechanisms including EGFR alterations, activation of alternative signalling pathways (e.g., AXL, HER3, STAT3), and downstream pathway reactivation. Similarly, resistance to cytotoxic agents such as cisplatin arises through mechanisms including enhanced DNA repair, altered drug accumulation, and molecular reprogramming [5].

Despite the identification of potential biomarkers in HNSCC, including viral (HPV, EBV), genetic (TP53, PIK3CA, NOTCH1), proteomic, and epigenetic markers, their clinical implementation remains limited. A major challenge lies in the lack of standardisation and robust validation across studies, with variability in detection methods, platforms and analytical protocols, leading to inconsistent and non-reproducible results [8].

Taken together, these limitations highlight the need for more integrative dynamic approaches capable of capturing the complexity and temporal evolution of HNSCC. Over the past decade, efforts have been made to develop computational models capable of addressing individual aspects of this disease, ranging from the modelling of tumour dynamics and treatment response [9,10] and the characterisation of drug exposure and pharmacodynamic effects [11] to the prediction of clinical outcomes and treatment decisions from patient cohorts [12,13]. However, these approaches do not constitute a platform that is continuously updated with multimodal patient data. For example, in cases where resistance mechanisms emerge after the start of treatment, models are needed that can be calibrated longitudinally to recalculate the best specific treatment pathway for that particular patient.

In this context, computational modelling, particularly Digital Twin (DT) frameworks, has emerged as a promising strategy to integrate multimodal patient-specific data and enable continuous simulation of disease progression and treatment response. Computational models in oncology have evolved from relatively simple mathematical descriptions of tumour growth and radiobiology to increasingly sophisticated, data-driven and multi-scale modelling frameworks with expanding applications in diagnosis, prognosis, and therapeutic planning [14]. The concept of DTs has emerged as a dynamic, patient-specific model that is continuously updated with real-world data, enabling the prediction of disease trajectories and supporting personalised treatment strategies, particularly in the context of targeted therapies, where predicting response and resistance remains a major challenge [15]. For oropharyngeal squamous cell carcinomas, DT-based approaches already exist, but none of them explores the molecular and pharmacodynamic dimensions [12,13]. To achieve this ideal, we propose that the architecture of a DT for HNSCC should incorporate a hybrid computational model capable of integrating multimodal data into a single model.

The proposed input data is divided into four core domains: imaging, molecular and multi-omic profiling, longitudinal liquid biopsy, and ex vivo functional testing. Imaging data is part of routine clinical practice, with images being collected from before the start of treatment and between therapy administrations. This data provide the basis for the DT, enabling the dynamics of the tumour (proliferation, cell density, hypoxia, amongst others) to be represented using biology-based mathematical models [9,10]. However, given the molecular heterogeneity characteristic of HNSCC, as well as the frequent emergence of resistance mechanisms during treatment, a model based solely on imaging fails to anticipate these developments. Thus, through a single multi-omic analysis, patients are molecularly stratified, defining the initial values of the model’s parameters and identifying the patient-specific alterations that will subsequently be monitored. Subsequently, using liquid biopsies, a minimally invasive and repeatable method, it would be possible to monitor these alterations longitudinally through the analysis of ctDNA and exosomes [16,17]. In this way, a layer is added to the model that enables more specific predictions of therapeutic resistance. The final domain is specifically designed for testing therapeutic agents and combinations, with a view to assisting in the calibration of the PK/PD models integrated into the DT. This domain is not continuously updated, but its main contribution lies in the initialisation of the model, as well as in preliminary testing of new drugs at the investigational stage. These categories are not intended to exclude other clinically relevant variables. They reflect different roles in model initialisation, longitudinal updating, and treatment evaluation.

In this review, we outline a conceptual framework for a DT in HNSCC in which multimodal data are integrated within a hybrid computational model. Such a DT could potentially improve patient stratification, anticipate the emergence of resistance, and support the design of adaptive and combination therapeutic strategies.

## 2. Literature Search and Selection

In this review, we conducted a literature search from February 2026 to August 2026 using electronic databases, including PubMed and Google Scholar. We used combined terms related to the disease (such as head and neck squamous cell carcinoma/HNSCC, head and neck cancer/HNC), to its molecular characterisation and treatment (such as molecular stratification, targeted therapy, EGFR, resistance mechanisms, PI3K, HRAS, biomarkers), to emerging monitoring tools (such as ctDNA, liquid biopsy, exosomes, organoids), to computational approaches (such as digital twin, mechanistic model, machine learning, PK/PD) and to challenges and limitations. Reference lists of retrieved articles were also screened for further relevant work. Priority was given to articles published within the last five years, except for foundational studies. Only articles written in English were accepted.

Regarding studies supporting the biology of HNSCC, resistance mechanisms and DT, no restrictive inclusion criteria were applied. For literature supporting DT technologies or other emerging computational approaches, priority was given to studies in patients with HNSCC or defined anatomical subgroups. When relevant to the topic, studies with other cancer types illustrating an approach not yet available in HNSCC were included. Only peer-reviewed articles were used, except for one preprint, which was included as it addressed interesting developments not yet covered by the peer-reviewed literature. This article and its limitations are highlighted where it is discussed.

Generative AI tools, ChatGPT (GPT-5.6 Sol, OpenAI) and Claude (Claude Sonnet 5 and Claude Opus 5, Anthropic) were used to assist with language refinement, grammatical correction, textual cohesion, and rephrasing of selected passages. We reviewed and edited AI-assisted outputs before inclusion in the manuscript. In the Figures, ChatGPT was used to generate the graphical element representing the DT, as indicated in the figure captions.

## 3. Molecular Stratification and Targetable Pathways in HNSCC

Molecular stratification in HNSCC is primarily defined by HPV status [18]. This distinction reflects fundamental differences in tumour aetiology, clinical behaviour, and therapeutic response. HPV-negative tumours are predominantly associated with exposure to carcinogens such as tobacco and alcohol and are characterised by poorer prognosis and increased resistance to therapy. In contrast, HPV-positive tumours are mainly associated with HPV viral infections, most commonly involving high-risk HPV-16, and are associated with a more favourable prognosis and improved therapeutic response [19,20].

When considering a DT applicable to HNSCC, we must be able to stratify patients at the molecular level. This need stems from several factors: (i) a markedly different prognosis depending on HPV status; (ii) variable responses to anti-PD-L1 immunotherapy; and (iii) the existence of subgroups with vulnerabilities that may be amenable to treatment (e.g., HRAS mutations, activation of the PI3K signalling pathway and cell cycle dependence) [21]. Although HPV status is a key feature for biomolecular stratification, it remains insufficient to capture the heterogeneity within HPV-defined groups or to effectively guide targeted therapeutic approaches.

At the molecular level, HPV-negative HNSCC displays greater genomic complexity, higher mutational burden and a broader spectrum of genomic alterations than HPV-positive tumours [22]. Among receptor tyrosine kinases (RTKs), EGFR is overexpressed in up to 90% of HNSCCs, leading to activation of downstream signalling pathways [23,24]. In particular, activation of the PI3K/AKT/mTOR pathway appears to be one of the most prevalent molecular events in HNSCC. Its high prevalence reflects the convergence of several independent mechanisms rather than a single recurrent alteration. EGFR-driven activation is the most common (47%), followed by PIK3CA mRNA overexpression (27.2%), PIK3CA amplification (14.2%), loss of the negative regulator PTEN (10–15%), and PIK3CA mutation (8.6%) [25]. Furthermore, whole-genome sequencing analyses have shown that nearly one third of HNSCC tumours harbour mutations in the PI3K signalling pathway, with the PIK3CA gene appearing as the most frequently mutated. In a subset of HPV-positive tumours, PIK3CA or PIK3R1 were the only mutated cancer- associated genes identified [26]. In addition, recurrent alterations affecting TP53, CDKN2A and CCND1 contribute to genomic instability and deregulation of cell-cycle control [27]. While these alterations define the molecular landscape of HNSCC, their translation into clinically actionable targets remains limited by interpatient heterogeneity, pathway redundancy, and dynamic tumour adaptation.

In addition to the PI3K/AKT/mTOR pathway, the RAS family of proto-oncogenes (HRAS, KRAS, and NRAS) emerges as another pathway with clinical relevance [28]. In HNSCC, although mutations in HRAS only account for about 4–5% of cases [29,30], they are one example of pathway-level stratification translating into clinical benefit. For example, tumours harbouring HRAS mutations are dependent on farnesylation for oncogenic signalling. The farnesyltransferase inhibitor Tipifarnib was therefore tested in patients with HRAS-mutant HNSCC. In a phase II trial, patients with a high HRAS variant allele frequency (VAF ≥20%) achieved an objective response rate of 55% and a median overall survival of 15.4 months with Tipifarnib, including in patients who had progressed on cetuximab, immunotherapy, or both [31]. These findings establish HRAS mutations as one of the few clinically actionable genomic alterations currently available in HNSCC, notwithstanding the very small fraction of the HNSCC patient population affected by these mutations and likely to benefit from HRAS-targeted therapy.

The Ras/RAF/MEK/ERK (MAPK) signalling cascade, due to its central role in cell proliferation, differentiation, cellular senescence, and drug resistance, is also a therapeutic target. Activating alterations affecting the MAPK pathway have been identified in approximately 1/5 of HNSCCs [32,33]. Significant differences in sensitivity to MEK inhibitors have been observed among HPV-negative HNSCC cell lines, suggesting that specific molecular subgroups may derive greater benefit from MEK-targeted therapeutic strategies [33].

The genomic diversity associated with this type of malignancy led to the development of transcriptomic classification systems designed to capture distinct tumour phenotypes. Among these, The Cancer Genome Atlas (TCGA) confirmed and refined previously defined four transcriptomic subtypes: basal, mesenchymal, atypical, and classical [22,34]. Although the TCGA transcriptomic classification remains the most widely used framework for molecular stratification in HNSCC, subsequent studies have further refined this classification, particularly within HPV-positive tumours, identifying additional immune- and keratinisation-associated molecular subgroups [35,36,37,38].

Despite these recurrent molecular alterations, translating pathway-level information into clinically useful predictive biomarkers remains challenging. This gap is particularly evident in the case of EGFR. Pharmacological strategies against EGFR span several mechanistic classes, including the monoclonal antibody cetuximab, small-molecule tyrosine kinase inhibitors, PI3K pathway inhibitors, and antisense gene-therapy approaches [39]. Although EGFR overexpression is common in HNSCC, especially in HPV-negative tumours, mutations in this gene are rare [39,40]. This is an important aspect that can determine the effectiveness of anti-EGFR therapies, because the mechanism by which these agents translate to benefit differs across tumour types. For example, in patients with non-small-cell lung cancer harbouring activating EGFR mutations (in China the prevalence was 38.4% and in Europe 14.1%), the use of an EGFR tyrosine kinase inhibitor improves the prognosis of these patients [41,42]. In colorectal cancer, the selection of anti-EGFR therapies depends on the status of the downstream RAS genes (KRAS and NRAS), and tumours carrying RAS mutations do not benefit from this treatment [43]. In HNSCC, EGFR overexpression does not reflect the same degree of pathway dependence, unlike what happens in the two previous examples, which helps to explain the limited and variable efficacy of cetuximab, the only approved anti-EGFR drug for this tumour [39]. High EGFR levels may reflect overexpression rather than true oncogenic addiction. Tumour cells may also rely on alternative receptor tyrosine kinases in order to maintain downstream signalling and survival.

Several candidate predictive biomarkers (e.g., PIK3CA/RAS mutations and loss of PTEN [44], caveolin-1/Sox-2 protein signature [45], treatment-induced reductions in phosphorylated EGFR (pEGFR) [46], serum VEGF/IL-6 [47]), have shown promising associations with treatment response, although their performance appears to be context-dependent and none has yet been validated for routine clinical use. Even the predictive biomarker currently used to guide anti-PD-1 therapy, PD-L1 combined positive score (CPS), provides only modest predictive value [48,49]. Beyond these examples, numerous diagnostic, prognostic and predictive biomarkers have been investigated in HNSCC. However, future studies are needed to establish standard protocols for their measurements and expression thresholds, as well as guidelines for their clinical interpretation, as highlighted by Eberly et al. (2024) [50].

More broadly, these biomarkers provide only a snapshot of the tumour at a single timepoint, usually before treatment begins, and, in fewer cases, during or after therapy. As such, this approach fails to capture changes in tumour signalling pathways that may continue to occur as a result of treatment. This underscores a substantial gap that dynamic, continuously updated computational modelling frameworks and DTs in particular aim to address. Within our conceptual framework, the multi-omic domain could assist in two functions: patient stratification through machine learning models at baseline and defining which patient-specific alterations should be monitored longitudinally through liquid biopsy.

## 4. Resistance Mechanisms and Adaptive Therapeutic Strategies

Resistance to targeted therapies and immunotherapy is one of the principal obstacles limiting durable treatment responses in HNSCC. Generally, resistance may be classified as intrinsic or acquired. Intrinsic resistance arises from pre-existing molecular or cellular characteristics that prevent an effective response from the outset of treatment, whereas acquired resistance develops after an initial therapeutic benefit through tumour adaptation under selective treatment pressure. Increasing evidence indicates that both forms of resistance coexist in HNSCC and are mediated by a complex interplay of genetic alterations, signalling network rewiring, tumour plasticity, clonal evolution, and interactions with the tumour microenvironment [51].

### 4.1. Pathway Reactivation and Bypass Signalling

In HNSCC, pharmacological inhibition of a receptor or signalling protein can be compensated for by the activation of parallel pathways or by the reactivation of downstream components, allowing tumour cells to maintain proliferation and survival. For example, cetuximab often exhibits limited clinical efficacy with receptor-level bypass emerging as one of the principal mechanisms underlying resistance. Yonesaka et al. (2011) [52] demonstrated that persistent ERBB2 signalling represents one example of this acquired resistance mechanism. Through either ERBB2 gene amplification or increased heregulin expression, the downstream ERK1/2 signalling pathways remain active despite EGFR inhibition, allowing tumour cells to maintain cell proliferation and survival. Importantly, inhibition of ERBB2 reverses cetuximab resistance. Consistent with these findings, ERBB2/HER2 overexpression has been reported in approximately 39% of untreated primary HNSCC tumours [53].

Alternative RTKs, including MET [54], AXL [55], and IGF-1R [56] receptors, can also be used in a similar way, all of which converge onto the same pathways of PI3K/AKT/mTOR and RAS/RAF/MEK/ERK that would have otherwise been triggered by the EGFR receptor.

Furthermore, the MET oncogene encodes the receptor tyrosine kinase c-MET, whose activation in cetuximab-resistant HNSCC has been associated with a characteristic epithelial-to-mesenchymal transition (EMT) phenotype, culminating in poorer prognosis through increased cell motility, invasion, and metastasis [57,58,59]. Dual inhibition of the EGFR and c-MET pathways has been shown to decrease MAPK and AKT phosphorylation, translating into reduced tumour growth and increased apoptosis [60]. Building on this rationale, a phase I trial evaluated the combination of cetuximab and ficlatuzumab to simultaneously target two pathways established as drivers of intrinsic tumour resistance, namely EGFR and the hepatocyte growth factor (HGF)/c-MET axis and found that simultaneous inhibition of these pathways was feasible and had the potential to partially overcome cetuximab resistance in recurrent or metastatic HNSCC, supporting further clinical evaluation [61].

It has also been shown in cetuximab-resistant HNSCC cells that AXL hyperactivation promotes upregulation of Src-family kinases and NRG1, a cascade that ultimately restores EGFR-HER3 signalling pathways downstream of the original blockade. AXL also appears to contribute to an EMT phenotype [55,62]. IGF-1R may also contribute to cetuximab resistance. In a study conducted by Iyer et al. (2016) [56], cetuximab-resistant cells showed a higher number of EGFR-IGF-1R heterodimers relative to sensitive cells, driving proliferative signalling via phosphorylation of EGFR at tyrosine 845. This phosphorylation was abolished by pharmacological IGF-1R blockade with picropodophyllin (PPP), confirming a causal role for IGF-1R in this resistance mechanism.

Beyond receptor-level bypass, tumour cells may also maintain survival through constitutive activation of downstream signalling cascades. Among them, the PI3K/AKT/mTOR pathway is the most frequently altered in HNSCC. Its constitutive activation may arise through several mechanisms, including PIK3CA mutations or amplification, loss of the tumour suppressor PTEN, or persistent stimulation by upstream RTKs, ultimately allowing tumour cells to maintain proliferation and survival despite EGFR inhibition [63]. Izumi et al. (2020) [64] used CRISPR/Cas9 genome editing to investigate how the PI3K/mTOR signalling pathway contributes to cetuximab resistance in HNSCC. The authors found that PTEN gene copy number loss, which negatively regulates PI3K signalling, was sufficient to sustain PI3K/mTOR pathway activity even when EGFR was pharmacologically inhibited. Loss of PTEN expression has been associated with poorer progression-free survival among patients treated with cetuximab, whereas patients with preserved PTEN and wild-type PIK3CA tend to derive greater benefit from treatment [63]. Dual inhibition of EGFR and PI3K/AKT pathway signalling has been evaluated as a therapeutic strategy. Early-phase studies of pilaralisib (SAR245408/XL147) and SAR245409 (XL765) combined with erlotinib in solid tumours showed acceptable tolerability but only modest antitumour activity [65,66,67]. In a randomised phase II trial, adding the PI3K inhibitor PX-866 to cetuximab provided no additional clinical benefit over cetuximab alone in patients with relapsed or metastatic HNSCC [68].

However, these trials did not select patients based on PI3K pathway alterations, which may explain the observed lack of benefit. Given that constitutive PI3K activation is restricted to molecularly defined subsets of HNSCC, biomarker-selected trials combining EGFR and PI3K inhibition may help identify subgroups more likely to benefit from combined EGFR and PI3K inhibition.

### 4.2. Tumour Plasticity and Clonal Evolution

HNSCC tumours can acquire resistance through mechanisms that do not rely on a single, static genetic event. Exposure of tumour cells to adverse metabolic conditions, various signalling pathways, and the stress of treatment itself creates an environment conducive to phenotypic changes, mediated primarily by epigenetic and transcriptional alterations. This behaviour is known as cellular plasticity and is similar to that observed in embryonic stem cells [69].

Among the different manifestations of tumour plasticity, EMT is one of the best-characterised and clinically relevant mechanisms in HNSCC [69,70,71], playing a role in the response to different chemotherapy treatments and targeted therapies. In non-small cell lung carcinoma cells that were closer to an epithelial state, a positive response to EGFR inhibitors was observed, whereas in a mesenchymal state, treatments with EGFR or PI3K inhibitors resulted in resistance; furthermore, these cells were found to have higher AXL expression and responded positively to inhibitors [72].

An incomplete EMT has also been shown to be a key factor in predicting poorer prognoses [70]. Puram et al. (2017) [73] analysed approximately 6000 transcriptomic profiles of individual cells derived from patients with oral cavity HNSCC. They divided the cell populations into malignant, stromal, and immune cells and found that, within the malignant cells, a distinct subpopulation stood out, exhibiting a partial epithelial-to-mesenchymal transition (p-EMT) phenotype. This intermediate population was located at the invasive edge of the tumour, close to cancer-associated fibroblasts. In vitro evaluation using the SCC9 cell line, derived from oral cavity HNSCC, identified a subpopulation showing a p-EMT profile similar to that identified in patient tumour. These p-EMT-high cells exhibited greater invasive capacity.

The impact of EMT on HNSCC is also evident at the cadherin level, with the occurrence of so-called “cadherin switching,” characterised by the loss of the epithelial adhesion molecule E-cadherin along with increased expression of the mesenchymal marker N-cadherin. This switch weakens cell-cell adhesion, while promoting motility and invasive behaviour. In HNSCC, cadherin switching has been associated with poorer histological differentiation, cancer cell growth, increased local invasion, and lymph node metastasis [74,75].

Moreover, activation of the EMT programme has been associated with the acquisition of cancer stem cell (CSC) characteristics through the loss of epithelial features, including cell-cell junctions, apical-basal polarity and epithelial morphology, together with the acquisition of migratory and invasive properties [76,77,78]. Accumulating evidence indicates that the relationship between EMT and stemness is more complex than initially proposed. Rather than representing equivalent biological programmes, EMT and stemness appear to constitute partially independent cellular states. Consequently, not all tumour cells undergoing EMT acquire CSC properties, while CSCs themselves may exist in epithelial, mesenchymal or intermediate (hybrid E/M) phenotypic states [79]. Owing to their plasticity and ability to adapt to changes in the tumour microenvironment, CSCs constitute a major therapeutic challenge. Although the mechanisms responsible for their resistance remain incompletely understood, these cells likely have enhanced DNA repair capacity, activation of anti-apoptotic pathways, increased expression of drug efflux transporters, acquisition of a quiescent state, adaptation to hypoxic niches, overexpression of stemness-related genes and evasion of immune surveillance [79,80].

Highlighting the fundamental role of the PI3K/AKT/mTOR signalling pathway, molecular regulators capable of activating the pathway (e.g., SLC4A7 [81], P4HA2 [82], STC2 [83] and SAR1A [84]) and leading to EMT progression were identified.

In parallel with tumour plasticity, clonal evolution represents another major mechanism underlying treatment resistance. High intratumour heterogeneity in HNSCC is characterised by the coexistence of multiple subclonal populations with distinct genetic and phenotypic profiles, including expression of transcriptional profiles related to stemness, proliferation, and EMT. Clonal evolution has been implicated in tumour recurrence, disease progression and treatment resistance [85,86]. An integrated multi-omics study demonstrated that cisplatin resistance was associated with the selective expansion of subclones harbouring gain-of-function TP53 mutations, together with activation of the PI3K/mTOR pathway [87]. Conventional single biopsies present inability to capture the full extent of this intratumour heterogeneity, potentially overlooking clinically relevant subclonal alterations [88]. A promising alternative is represented by liquid biopsies through circulating tumour DNA (ctDNA) analysis combined with whole-exome sequencing (WES), allowing tumour evolution to be monitored throughout treatment. In a study of patients with locally advanced HNSCC, Bruixola et al. (2025) [89] demonstrated that ctDNA analysis identified additional oncogenic variants not detected in tissue, accounting for up to 67% of all variants detected. Moreover, ctDNA profiling revealed resistance-associated alterations affecting the PI3K-mTORC2 and ATM-CHK2 signalling pathways during disease progression.

### 4.3. Immune Escape and Resistance to Immunotherapy

Despite the growing use of immune checkpoint inhibitors (ICI) in HNSCC, the mechanisms underlying resistance to immunotherapy remain only partially understood. Clinically, resistance can be classified into three categories: primary, in which patients gain no initial clinical benefit from treatment; adaptive, in which the tumour is recognised by the immune system but protects itself through active adaptation; and acquired resistance, in which an initial beneficial response takes place, but it is followed by disease progression [90].

From a biological perspective, the mechanisms underlying resistance can be broadly divided into tumour cell-intrinsic and tumour cell-extrinsic processes. Tumour cell-intrinsic mechanisms include the absence of recognisable tumour antigens, defective antigen presentation as a consequence of mutations in HLA class I or β2-microglobulin alterations, disruption of IFN-γ signalling, oncogenic pathway activation (e.g., MAPK, PI3K/AKT and WNT/β-catenin), constitutive PD-L1 expression, and reduced susceptibility to cytotoxic T-cell-mediated killing. Tumour cell-extrinsic factors, on the other hand, include deficiency of T cells, compensatory upregulation of inhibitory immune checkpoints (e.g., LAG-3, TIM-3 and VISTA), and the recruitment of immunosuppressive cell populations, including regulatory T cells, myeloid-derived suppressor cells and tumour-associated macrophages, all of which contribute to immune evasion and therapeutic failure [22,90,91,92].

Consistent with the HPV-based molecular stratification discussed previously, immune resistance mechanisms also differ according to HPV status. Saloura et al. (2017) [93] reported that HPV-negative tumours exhibit greater clonal expansion of tumour-infiltrating T cells with high levels of HLA-A expression compared with HPV-positive HNSCC. This indicates that HPV status, already relevant for molecular stratification, may also influence immunotherapy response.

### 4.4. From Static Biomarkers to Dynamic Patient Modelling

Throughout this chapter, we have explored the best-known mechanisms of resistance in HNSCC and demonstrated that a patient’s response to HNSCC treatment results from a complex network of ongoing interactions between tumour-intrinsic and tumour-extrinsic processes, rather than from a single molecular alteration. The five discussed groups (pathway reactivation, activation of bypass signalling, tumour plasticity, clonal evolution, and immune escape) coexist and evolve throughout treatment under therapeutic selective pressure, progressively reshaping tumour biology.

The main clinical biomarkers currently available to support therapeutic decisions, such as HPV status and PD-L1 CPS, together with molecular alterations such as PIK3CA mutations, are generally evaluated before treatment and provide a static representation of the tumour. They do not inform about the molecular and phenotypic changes that may occur during therapy, including the emergence of resistant clones, activation of alternative signalling pathways, changes in the tumour microenvironment, and transitions between epithelial and mesenchymal cellular states. Hence, their predictive value for selecting the optimal therapeutic approach may be decreased as the tumour adapts to the treatment itself.

A promising alternative involves the longitudinal evaluation of new biomarkers, either through ctDNA, which enables non-invasive assessment of clonal evolution and the emergence of resistance-associated genomic alterations throughout treatment, circulating extracellular vesicles, proteomic signatures, or immune-related biomarkers. A dynamic assessment of the patient provides complementary information regarding tumour-host interactions, whereas serial imaging and radiomic analyses may capture spatial and temporal changes in tumour phenotype that preceded conventional radiological progression.

However, simply having all this longitudinal data is not enough. This information must be integrated to allow a better understanding of the complex biological interactions underlying each HNSCC patient. Significant recent advances in computational modelling and artificial intelligence (AI) could allow researchers to integrate this heterogeneous multimodal clinical data into dynamic models that mirror the patient’s unique characteristics. In particular, the potential applications of DTs in oncology enable us to optimise patient-centred treatment decisions, whether the treatment plan is based on radiation therapy, chemotherapy, immunotherapy, or a combination of different strategies.

The potential translation of these resistance mechanisms into longitudinal monitoring strategies and DT-guided therapeutic adaptation is presented in Table 1 (Section 6).

## 5. Computational Modelling and Digital Twin Frameworks

The biological complexity described above requires computational models capable of representing tumour evolution across multiple spatial and temporal scales. DTs have emerged as one of the most promising frameworks for achieving this objective. The first applications of DTs date back to engineering, where the concept refers to a system consisting of a physical product, its virtual counterpart, and the bidirectional exchange of data and information between the two parts [94,95]. More recently, this technology was recognised by the National Academies of Science, Engineering, and Medicine as a technology with high predictive potential, as reflected through the exponential growth in the number of scientific publications in recent years [96]. The application of this technology in healthcare holds considerable value, particularly in oncology, given the heterogeneity and variability of tumour behaviour during treatment [97].

What sets a DT apart from other predictive models is its longitudinal updating, based on continuous two-way communication between the physical component and the virtual component. The patient provides the model with clinical, genomic and monitoring data at different time points, and the virtual component, by integrating this data, enables a personalised treatment or intervention to be determined with each update [98].

For the purposes of this review, we define an ideal DT for HNSCC as a model based on four essential criteria: (i) the model must be patient-specific and calibrated with patient data; (ii) from baseline and throughout the treatment process, the DT should be fed with new patient data; (iii) as the model is updated, the DT must be able to provide new, updated outputs (bidirectional coupling); and (iv) the model should be able to simulate different therapeutic scenarios for the patient and compare their predicted outcomes [97]. These four criteria are consistent with the 5Is proposed by Katsoulakis et al. (2024) [96], who describe a DT as a platform that is “individualized, interconnected, interactive, informative, and impactful”. To meet these criteria, the DT framework typically comprises a data layer that consolidates the patient’s multimodal data; a computational layer that processes this data through hybrid models combining mechanistic modelling and machine learning, enabling simulation and prediction; and an interface layer that translates model outputs into clinically interpretable decision support tools [97].

Within this framework, population-based datasets must be incorporated to estimate the model structure, parameter distributions and prior probabilities [10]. When applied to a patient, imaging and molecular data can be used to initialise or calibrate the patient-specific model. As new longitudinal data become available, these parameters can be progressively recalibrated [10,99]. If Bayesian approaches are employed, this updating may also be used to estimate posterior parameter distributions and their associated uncertainty [99].

This architecture translates into a crucial predictive capability for simulating disease progression and anticipating responses to chemotherapy, immunotherapy or radiation, or a combination of these, allowing the clinicians to make decisions that maximise therapeutic value and efficacy, improve survival rates and quality of life [98].

Molecular stratification based on HPV status, recurrent genomic alterations (e.g., *PIK3CA* and *HRAS* mutations), EGFR expression, or PD-L1 CPS provides valuable information for patient selection. Still, it fails to capture the continuous biological adaptations that occur during treatment, including pathway reactivation, clonal evolution, and the emergence of resistance. To our knowledge, no current model integrates molecular, imaging, and functional data within a single, continuously updated simulation of the patient’s response to personalised therapy.

Current approaches toward HNSCC DTs draw on a spectrum of computational architectures. These include (i) mechanistic formulations, (ii) pharmacokinetic/pharmacodynamic (PK/PD) models, (iii) data-driven machine learning, and (iv) hybrid networks.

Each computational system has different advantages and disadvantages, but their integration provides this framework with powerful capabilities for processing information from the four different data domains we propose.

Mechanistic models enable us to represent tumour dynamics by applying biology-based mathematical equations to parameters derived from imaging data. These parameters can be updated longitudinally using patient data, allowing the model’s predictions to adapt over time [9,10]. In breast cancer, the application of this type of model has shown potential for predicting pathological complete response and optimising patient-specific treatment regimens [99,100]. However, current imaging-mechanistic models remain limited in their ability to incorporate molecular information that evolves over time and to dynamically represent resistance mechanisms.

In turn, PK/PD models enable the relationship between drug exposure and the observed biological effect to be characterised by incorporating patient-specific clinical and pharmacological data [11]. Within a DT, their integration could enable optimisation of the dose and treatment regimen based on each patient’s pharmacokinetic profile [101]. When used in isolation, these models do not fully account for the decline in drug sensitivity that may occur as resistance mechanisms emerge over time.

Data-driven machine learning models, through the incorporation of multi-omic data, can complement the two previous approaches and incorporate new molecular or liquid biopsy data. In this way, they can support not only patient stratification but also the identification of patterns associated with the emergence of resistance mechanisms [102,103,104]. However, these models require attention to biological interpretability and representative training cohorts [105,106]. We suggest that the inclusion of an explainable artificial intelligence (XAI) interface could aid the interpretation of the model’s outputs, as illustrated by the DITTO model discussed in Section 6.6 [13].

For this reason, a hybrid architecture is proposed to simultaneously represent spatial tumour dynamics, drug exposure, high-dimensional molecular data, and a clinical decision interface, as it allows each component to perform the task for which it is best suited.

## 6. Towards an Integrated Digital Twin for HNSCC: A Framework Perspective

**Figure 1:**
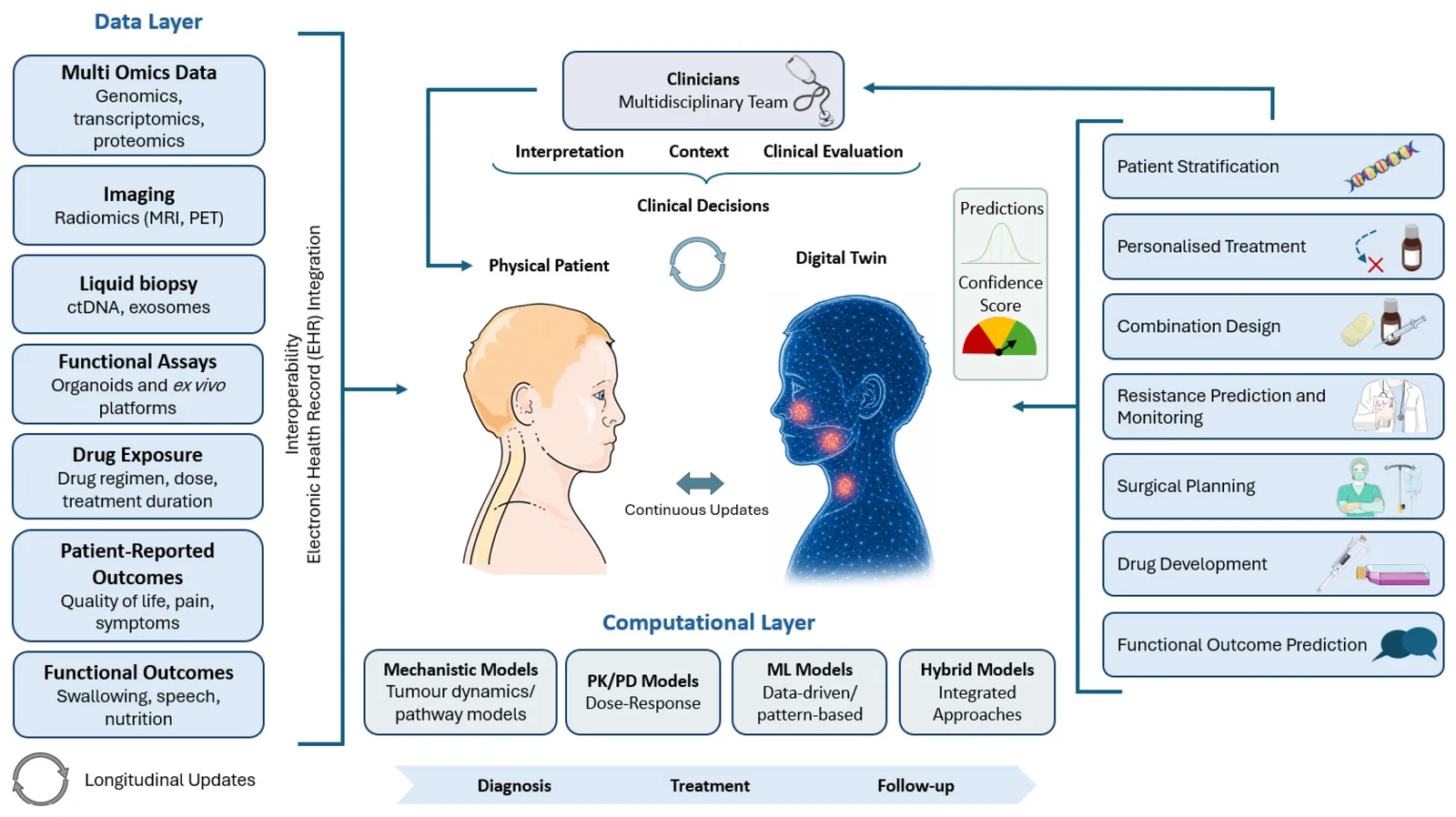
Schematic representation of a Digital Twin (DT) framework for HNSCC. Multimodal patient-specific data are acquired at baseline and/or longitudinally and integrated into a computational framework combining mechanistic, PK/PD, and machine learning models within a hybrid architecture. In the proposed framework, baseline multi-omic data could define the initial molecular state and identify patient-specific alterations to be monitored, including PIK3CA or HRAS mutations. Longitudinal imaging could update tumour dynamics, while liquid-biopsy data could provide information on evolving molecular changes associated with emerging resistance. Functional assays could inform drug sensitivity and pharmacodynamic response, whereas treatment and drug-exposure data could support PK/PD modelling. Patient-reported and functional outcomes could provide additional longitudinal information on treatment tolerability, symptoms, and functional impact. As new patient data become available, these inputs could be used to recalibrate the patient-specific model and update its predictions over time. When different data modalities provide discordant information, this discordance could be reflected in the uncertainty of the model predictions and may prompt additional data acquisition or clinical review before treatment adaptation. Predictive uncertainty is conceptually represented by the confidence score (right side of the DT). The illustrative red-orange-green colour gradient indicates increasing confidence. The multidisciplinary team would remain responsible for interpreting, contextualising, and clinically evaluating the model outputs before making a clinical decision. Through this iterative feedback loop, the DT evolves alongside the patient and could support dynamic prediction of tumour behaviour, molecular stratification, personalised treatment selection, optimisation of combination therapies, response and resistance monitoring, surgical planning, drug development, and clinical decision-making. In the future, an ideal HNSCC model could be integrated into clinical systems to support personalised treatment and multidisciplinary decision-making. Graphical elements were obtained from Servier Medical Art (https://smart.servier.com, accessed on 30 August 2026), licensed under CC BY 4.0 (https://creativecommons.org/licenses/by/4.0/, accessed on 30 August 2026), except for the graphical element representing the DT, which was generated and edited with the assistance of the artificial intelligence (AI) tool ChatGPT, version GPT-5.6 Sol (Open AI).

To develop a potential hybrid computational model for a DT in HNSCC, we propose the integration of four data domains: (i) imaging, providing information on tumour dynamics; (ii) multi-omics, including genomics to support molecular stratification and identify relevant alterations for monitoring; (iii) liquid biopsy data, including ctDNA and exosomes, to enable longitudinal monitoring of molecular changes and emerging resistance; and (iv) ex vivo functional testing, as an alternative method for assessing drug response and supporting treatment evaluation (Figure 1).

### 6.1. Imaging and Mechanistic Modelling

Among the approaches that are already well established in the literature, imaging-based models have been extensively investigated in HNSCC as tools for predicting response to radiotherapy. These models are based on quantitative and multimodal imaging. This data is crucial because it allows us to capture tumours’ individual biological heterogeneity by estimating critical parameters, such as the growth fraction, cell density, and cell cycle time, thereby accurately predicting each patient’s therapeutic response [9].

In fact, Aliotta et al. (2025) [9] developed a mechanistic radiobiological model for patients with HPV-positive oropharyngeal head and neck cancer (HNC) to predict and monitor the specific response to radiotherapy. In their study, they integrated structural and functional data from diffusion-weighted magnetic resonance imaging (DW-MRI) with metabolic and hypoxia biomarkers from positron emission tomography (PET) ([18F] fluorodeoxyglucose (FDG) and [18F] fluoromisonidazole (FMISO)). Initially, patient-specific imaging studies were integrated to enable longitudinal mapping of the macroscopic tumour volume throughout treatment. This type of modelling proves useful in developing an optimal DT for HNSCC due to its longitudinal updating, which is achieved through the continuous incorporation of new data during treatment. Furthermore, the model’s predictive accuracy increased substantially after the inclusion of data from the first week of treatment. Importantly, this personalised model outperformed the generic population-based model in predicting tumour regression and was shown to provide useful clinical information two weeks earlier [9].

Another independent study demonstrated the same predictive potential in models using different imaging modalities (diffusion-weighted imaging (DWI), MRI, and FDG-PET/CT). It is worth noting that the functional changes observed during radiation therapy have similar predictive value for treatment response and disease recurrence [107].

More recently, Hormuth II et al. (2026) [10] applied a similar mechanistic approach to a cohort of 20 patients with HPV-associated oropharyngeal carcinomas. By analysing oxygen-enhanced MRI (OE-MRI) acquired at baseline and during treatment, they were able to assess hypoxia, together with perfusion and cellularity, derived from dynamic contrast-enhanced MRI (DCE-MRI). Based on these parameters, they were able to subdivide the tumours into four distinct microenvironments and understand their dynamics throughout chemoradiotherapy using a system of coupled differential equations. The model comprises a set of parameters (proliferation rates, transition between habitats, reoxygenation rate and radiosensitivity) calibrated using 75% of the cohort, and subsequently applied to held-out patients, whose models were initialised using their individual baseline habitat composition. In breast cancer, a similar approach based on MRI segmentations, diffusion-weighted MRI and DCE-MRI has made it possible to assess, respectively, the nature of the tissue (tumour, fibroglandular and adipose), cell density and pharmacokinetic profiles, including the spatial distribution of the drug, thereby enabling the prediction of response to neoadjuvant chemotherapy and the personalisation of the treatment regimen, based on parameters calculated from the images (proliferation rate, tumour cell mobility, and the efficacy and decay of each drug) [99,100].

However, these modelling studies have similar methodological limitations, namely small cohorts, lack of external validation, and no quantification of predictive uncertainty (possible by using Bayesian methods), which, for now, prevent their direct translation into clinical practice.

Additionally, they do not account for the molecular heterogeneity of the tumour or the associated resistance mechanisms. However, despite advances, current mechanistic models based on imaging capture where the tumour is and how it changes but remain unable to capture the continuously evolving molecular landscape of HNSCC. To remain predictive as resistance evolves, it would need to incorporate the extensive signalling redundancy that enables EGFR inhibition to be bypassed through alternative RTKs, including ERBB2, MET, AXL, and IGF-1R.

### 6.2. Genomic and Multi-Omic Stratification

An ideal HNSCC DT must be attached to the genomic and multi-omic stratification already established throughout this review, including HPV status, TP53/CDKN2A alterations, PIK3CA mutations, together with the pathway redundancy also discussed. Here, stratification has relied predominantly on data-driven machine-learning approaches capable of resolving molecular heterogeneity. Genomic data, however, only partially capture tumour heterogeneity. Multi-omic integration, combining genomic, transcriptomic, and proteomic layers, allows a resolution that neither imaging nor genomics alone can achieve.

This is illustrated by Wu et al. (2025) [102], who, in a multi-omic study on oral cavity squamous cell carcinoma (OSCC), identified A3A and EGFR as inversely expressed markers critical for patient stratification. High A3A (or PD-L1) expression correlated with favourable responses to anti-PD-1 therapy. Conversely, high EGFR-expressing tumours featured chr.7p11.2 gains and DNA repair alterations in patient-derived xenograft (PDX) models. These tumours responded to cisplatin plus cetuximab, whereas high-A3A tumours did not. This reciprocal axis highlights how multi-omic profiling could guide precise anti-PD-1 or anti-EGFR decisions and how powerful it would be to incorporate such strategies in a DT for HNSCC. Additionally, RRAS has emerged as a novel prognostic marker for local recurrence. Cell-based and clinical data showed that RRAS drives resistance to radiotherapy and cisplatin via EGFR/RRAS/AKT/ERK pathway activation and AKT/ERK phosphorylation. Interestingly, despite promoting recurrence, high-RRAS tumours demonstrated better clinical outcomes when treated with cetuximab.

Another illustrative example is the work by Liu et al. (2025) [103], who integrated single-cell and bulk tissue RNA sequencing data from ten independent datasets, totalling more than 59,000 cells, to identify three molecular subtypes of HNSCC with distinct prognostic implications. The subtypes differed significantly in terms of survival, the rate of genomic alterations (including TP53 mutations), the composition of the immune microenvironment, and, critically, sensitivity to chemotherapeutic agents such as 5-fluorouracil and docetaxel, as well as response to immunotherapy. The authors also developed a machine-learning-based clinical risk model, validated across four independent cohorts, capable of distinguishing high- and low-risk patients based on ten key genes.

More recent evidence points toward richer multi-omic DT models developed specifically for HPV-associated disease. However, as the authors point out, the model “remains a statistical and computational abstraction of tumour biology rather than a complete physiological replica” [108].

Together, these findings underscore that multi-omic integration is not merely an additional layer of information but a fundamental requirement for developing clinically meaningful DTs capable of supporting precision treatment in HNSCC.

### 6.3. Longitudinal Liquid Biopsy: ctDNA and Exosomes

Whereas the multi-omic profiling described above provides a comprehensive yet static snapshot of tumour biology, ctDNA enables this molecular profile to be monitored longitudinally through minimally invasive sampling. Among the longitudinal markers that a DT could incorporate, ctDNA is arguably the most extensively validated in HNSCC and can be obtained via the non-invasive method of liquid biopsy. Their integration into computational frameworks capable of continuously updating patient-specific DTs remains largely unexplored in HNSCC. ctDNA is attractive due to its potential for early detection and its ability to provide insights into tumour burden and mechanisms of resistance. One of the most widely studied markers is methylation [109]. Schröck et al. (2017) [110] measured plasma SHOX2 and SEPT9 methylation in a prospective observational cohort and reported a sensitivity of 52% and a specificity of 95% for the diagnosis of HNSCC, with higher methylation levels correlating with a poorer outcome. Other studies had already demonstrated the potential predictive value of ctDNA monitoring for resistance. Braig et al. (2016) [111] showed that, following cetuximab treatment, acquired KRAS, NRAS or HRAS mutations could be detected in over a third of patients. In approximately half of the cases, these mutations were identified as early as 16 weeks before clinical disease progression. Furthermore, in a more recent study, ctDNA analysis using whole-exome sequencing revealed alterations involving the PI3K-mTORC2 and ATM-CHK2 signalling pathways during disease progression [89].

For asymptomatic colorectal cancer, the US Food and Drug Administration has approved the SEPT9 methylation test in blood, the first blood-based marker for this type of cancer [112,113]. In breast cancer, using sequential targeted ctDNA analyses (every 6 months over a period of 4 years), it was possible to predict cancer recurrence up to 2 years in advance [114].

Although ctDNA represents a promising source of information, only around 0.01–5% of cell-free DNA (cfDNA) consists of ctDNA. Its analysis requires ultra-sensitive targeted techniques, including droplet digital PCR (ddPCR), BEAMing, and real-time PCR. When applied to pre-identified mutations, these techniques enable a low-cost, rapid and sensitive assessment. Conversely, untargeted approaches require larger input volumes and entail higher costs. Extra care is also needed during sample processing to reduce wild-type DNA noise, notably by ensuring the protocol is carried out swiftly, maintaining defined temperatures, performing double plasma centrifugation, and using specific kits for cfDNA collection [16,115,116,117]. Before any of the approaches described above could be applied into a DT, it is necessary to standardise and validate the ctDNA processing methods for clinical use [16]. Ideally, early identification of variants of interest in the primary tumour by NGS, as demonstrated in the study by Coombes et al. (2019) [114], would enable a more focused subsequent analysis of patient-derived ctDNA. Beyond predicting relapse, ctDNA may also provide information on the emergence of resistance mechanisms [118].

Exosomes provide a complementary source of information. These nano-sized, membrane-bound extracellular vesicles carry proteins, lipids and genetic material (including microRNAs) from their parent tumour cells, and can modulate the surrounding tumour microenvironment through cell-to-cell communication [119]. Owing to their stability in bodily fluids and the fact that they represent their parent cells, they have attracted particular attention as liquid biopsies [17,120]. Exosomes in HNSCC patients negatively modulate the immune environment, e.g., by suppressing T-cell activation and proliferation and inducing apoptosis in CD8+ cells, allowing the progression of cancer in patients to be inferred from the extent to which immune cell functions are suppressed [121,122]. In patients with tumours at more advanced stages, exosomes carrying active PD-L1, which suppresses T-cell function, have already been identified [123]. Another potential marker involves the assessment of microRNAs (miRNAs) present in exosomes as a predictive tool for tumour progression. In oral squamous cell carcinoma, miR-21 levels have also been shown to be associated with the promotion of metastasis. Their levels correlate with altered immune response, lymph node metastasis, as well as HIF-1α/2α expression [124]. These findings have also been corroborated in two other types of cancer, namely laryngeal [125] and oesophageal [126] cancers.

However, there are a number of challenges that need to be overcome before their application in monitoring can be translated into a DT framework. These range from the heterogeneity of exosomes (size, origin and molecular diversity) to the isolation and preservation protocols themselves. As reviewed by Dikici et al. (2025) [127], there is as yet no universally accepted protocol that allows for the simultaneous achievement of high yields and high purity. For example, differential ultracentrifugation, whilst being a cheap and easy-to-implement method, can result in samples co-sedimenting with proteins and lipoproteins; immunoaffinity capture, a rapid and efficient method, is expensive, cannot be applied to large volumes and requires expertise on the part of the researcher; microfluidics, whilst a method that combines efficiency, speed and high purity levels, is offset by the high costs and complexity of the equipment used. Furthermore, exosomes present additional challenges in terms of preservation (due to their short half-life and tendency to aggregate), and to date there is no standardised protocol [127,128]. For these reasons, the longitudinal monitoring of exosomes in a DT will require standardised protocols, as well as analytical validation of the isolation and quantification workflows.

### 6.4. Ex Vivo Functional Testing and PK/PD Integration

A second major objective in developing robust treatment algorithms for HNSCC is to characterise how exposure to individual therapies or their combinations influences tumour biology and therapeutic response. Clinically, current standard treatments are based on a combination of surgery (where possible) with platinum-based chemotherapy and radiotherapy, cetuximab, or the use of checkpoint inhibitors such as pembrolizumab and nivolumab, depending on the tumour grade, location, HPV status and PD-L1 scores [6,129]. However, once therapy has begun, treatment is guided by standardised dose-response assessments, with adjustments made primarily in response to treatment-induced toxicity or, in other cases, on the basis of radiological imaging showing tumour progression [129]. Consequently, clinicians have limited means of anticipating how differences in drug exposure influence tumour response, or even how resistance progressively alters the pharmacodynamic efficacy of a given regimen.

PK/PD models can help elucidate the relationship between exposure (PK), response (PD) and biomarkers for a given treatment. However, to develop these mechanistic models, preliminary studies are required to generate the initial simulations. In HNSCC, these foundations can be established using ex vivo testing, for example, in organoids, or even in vivo using xenografts [130]. In that sense, Millen et al. (2023) [131] generated a set of organoids derived from HNC (counting HNSCC and salivary gland adenocarcinomas) patient tumour tissue and assessed their response to chemotherapy, radiotherapy and even potential future therapeutic targets [131]. By correlating these organoids with their genetic background and response to different therapies, and integrating them into a PK/PD model, fundamental insights can be gained, paving the way for the discovery of new therapies or combinations. Besides, HNSCC organoids can recapitulate key features of the initial tumour and, in drug sensitivity tests, have been shown to differ from 2D cell lines, more closely approximating the responses observed in vivo [132]. In 2025, a biobank of organoids was established specifically to aid in patient stratification, as well as to support the evaluation of therapies [133].

However, organoid models have certain limitations that must be taken into account. The establishment of organoid models is a labour-intensive and time-consuming process, with variable success rates depending on the type of tumour and sample [131,134,135]. For example, in organoids established from metastatic colorectal cancer by Ooft et al. (2019) [135], the success rate was 63%, whilst in HNC, success rates were 28.4% in primary tumour samples and only 4.5% in metastatic samples [131]. To address this issue, a multiple-core biopsy, followed by assessment by a pathologist, may be a solution [131,135]. In the study by Millen et al. (2023) [131], amongst the tumour samples assessed by a pathologist, those containing tumour epithelial cells had an organoid establishment success rate of 85.5%, compared with 33.3% overall. This result is consistent with the correlation between the cellularity of the parental biopsy and culture success reported by Vlachogiannis et al. [136]. Another relevant aspect warranting caution concerns the correlation between the response of the organoids and that of the patient, due to the absence of the tumour microenvironment. The absence of stroma and an immune component influences the response to chemotherapy and radiotherapy [131,135,137]. In the case of radiotherapy, this treatment induces immunogenicity through antigen presentation [137]. Therefore, immune cells are likely required to capture its full effect; however, in conventional epithelial-only organoids, neither post-radiotherapy immune-mediated tumour cell killing nor the effect of immunotherapy on regional lymph nodes can be assessed in such assays [131].

More complex approaches have sought to recapitulate some of the components of the tumour microenvironment, including air-liquid interface (ALI) culture models and microfluidic 3D culture, such as patient-derived organotypic tumour spheroids. In these models, stromal and immune components can be preserved, at least partially [138]. Neal et al. (2018) [139] generated ALI patient-derived tumour organoids, and they found that these models preserved some native immune populations, such as T cells, B cells, NK cells and macrophages, together with cancer-associated fibroblasts/myofibroblasts. These organoid models also conserved the T-cell receptor repertoire of the parental tumour and demonstrated functional responses following blockade of the PD-1/PD-L1 immune checkpoint.

Ex vivo functional assays can provide information on tumour sensitivity, while PK/PD modelling can help us understand drug exposure and how it may influence treatment response. In HNSCC, the prospective CetuxIMAX study conducted by Marin et al. (2026) [11] followed HNSCC patients treated with cetuximab-based therapy to understand whether plasma cetuximab concentrations could have predictive value and to characterise cetuximab pharmacokinetics using a population pharmacokinetic model. By integrating plasma concentration data collected before and after cetuximab infusion at different treatment time points into the model, they observed that drug exposure varied considerably between patients (>40%) and that early plasma cetuximab concentrations were shown to predict disease control. This study, although not a DT, highlights how longitudinal drug-exposure data obtained from blood samples can be incorporated into a PK model. In a future DT, the application of similar methodologies, combined with longitudinal measurements of tumour response and resistance, could provide a more comprehensive representation of tumour biology during treatment and help characterise the exposure–response relationship.

### 6.5. Toxicity Prediction

Given the side effects associated with therapeutic regimens in HNSCC, it is important to minimise their functional impact on speech, swallowing, and breathing. Normal Tissue Complication Probability (NTCP) models have already been developed to predict treatment-related toxicity. In HNSCC, these models focus particularly on predicting dysphagia (difficulty swallowing) and xerostomia (sensation of dry mouth due to reduced saliva production) following radiotherapy. These models typically incorporate the dose received by organs at risk (parotid glands, submandibular glands, pharyngeal constrictor muscles), together with clinical data, and patient characteristics [140,141].

de Vette et al. (2025) [141] developed a deep-learning model that used 3D dose information, computed tomography (CT) scans, and organ-at-risk segmentations to predict late-onset dysphagia after radiotherapy. The model also provides attention maps, which indicate to the user the anatomical regions in the image that contribute to the predictions, thereby potentially increasing user confidence in the model outputs. When compared with conventional NTCP models, the proposed approach achieved higher predictive performance. Similarly, Men et al. (2019) [142] developed a deep learning model that integrated treatment-planning CT images, 3D dose distributions, and parotid and submandibular gland contours to predict radiotherapy-induced xerostomia in patients with HNSCC. This approach was subsequently extended by Chu et al. (2025) [143], who incorporated additional organ-at-risk segmentations and clinical variables. Both models demonstrated potential predictive capability compared with the conventional NTCP models [142,143].

The Comprehensive Individual Toxicity Risk Profile (CITOR), developed by Van den Bosch et al. (2021) [144], aimed to address this fragmentation by simultaneously evaluating six clinical domains (swallowing, salivary function, mucosa, speech, pain, and general condition). The profile was built from logistic-regression models predicting 22 toxicities across ten follow-up time points in a development cohort of 750 patients, and was subsequently validated in an independent multi-centre Dutch cohort.

Although NTCP modelling has been developed primarily in the context of radiotherapy, toxicity prediction remains directly relevant to targeted therapy, given that combination regimens involving EGFR or ICI with radiotherapy compound the risk of overlapping toxicity. Taken together, these studies suggest that integrating toxicity prediction into patient-specific DTs could enable simultaneous optimisation of tumour control and treatment-related morbidity, rather than predicting individual toxicities in isolation.

The proposed DT must be able to balance predicted tumour response against treatment-related toxicity. For example, if a therapeutic strategy is predicted to provide favourable tumour control but carries a high risk of severe toxicity, it should be excluded or deprioritised. The DT must be able to identify an alternative therapeutic regimen with an acceptable trade-off between tumour control and toxicity. For instance, when developing a DT for triple-negative breast cancer using a biology-based mathematical model, Christenson et al. (2025) [99] constrained the optimisation process so that therapeutic options with estimated toxicity exceeding that estimated for the standard-of-care treatment were not selected. To estimate toxicity values, they relied on drug concentration-time profiles. However, prospective clinical validation of such toxicity-constrained DT optimisation remains necessary before its application to HNSCC. Within the conceptual framework for HNSCC, patient-derived tumour organoids could also contribute to this balance by providing information on tumour sensitivity to alternative therapeutic regimens.

### 6.6. Emerging Applications of Digital Twins in Head and Neck Cancer

Although no comprehensive model currently combines dynamic and multimodal approaches, recent studies have established key methodological foundations that bring the development of a clinically relevant DT for HNSCC closer to reality. Tardini et al. (2022) [12] applied and evaluated the first use of a deep Q-learning DT dyad as a predictive tool for assessing survival and toxicity in treatments administered to patients with oropharyngeal squamous cell carcinoma. The framework comprises a “digital patient” model, which predicts tumour and toxicity responses at each stage of treatment, and a “digital physician” model, which predicts the treatment decisions a clinician would make. Both models were trained using data from a cohort of 536 patients who underwent definitive radiotherapy at MD Anderson Cancer Center. Building directly on this framework and using the same patient cohort, Wentzel et al. (2024) [13] introduced DITTO, a visual computing system based on a DT for the treatment of oropharyngeal cancers. This tool models the three standard-of-care treatment decisions: induction chemotherapy, concurrent chemotherapy, and neck dissection, and, at each stage, predicts both tumour response (locoregional control, distant metastasis, and overall survival, via deep survival machines) and toxicity risk (dose-limiting toxicities, feeding tube dependence, aspiration), alongside a “policy model” that recommends the next treatment step, balancing tumour eradication with the reduction of side effects. This interface uses a deep reinforcement learning model, enabling the prediction of short- and long-term disease outcomes and the risk of toxicity from a given treatment for that patient. This model also incorporates an explainable AI (XAI) method, allowing oncologists to visually understand the rationale underlying the model’s recommendation, thereby strengthening clinical confidence.

A recent preprint by Islam et al. (2026) [104] demonstrated how a machine learning-based DT has opened up new avenues in the molecular stratification of HNSCC. This model combined harmonised genomic, clinical and demographic data from 1046 patients obtained from the Oncology Research Information Exchange Network (ORIEN). The authors showed that twin-defined subgroups differ in terms of comorbidity burden, survival and underlying gene expression, and that survival models restricted to a patient’s own matched cohort outperformed models trained on the entire population. This study, while reinforcing the points detailed previously, also adds that data on chronic obstructive pulmonary disease, cardiovascular diseases and hypertension need to be integrated alongside tumour-intrinsic pathways for an HNSCC DT to reflect true patient heterogeneity. As a non-peer-reviewed preprint with small subgroup sizes and no correction for multiple comparisons, these findings remain hypothesis-generating, but they illustrate concretely what an ideal DT stands to gain from combining these data layers rather than treating them separately.

Among these approaches, DITTO is currently the model that most closely resembles a DT, but it does not yet include the molecular context (PIK3CA, HRAS or EGFR signalling pathways), nor does it incorporate radiological imaging, ctDNA or exosome markers. The system that comes closest to an operational, multi-outcome DT for HNSCC is, therefore, also the clearest illustration that clinical-level decision support and molecular-level tumour biology have not yet been integrated into a single framework.

**Table 1:** Comparison of principal studies and emerging approaches relevant to DT development in HNSCC. For each patient-specific and DT-like computational model, we indicate which of the DT criteria outlined in Section 5 are met or partially addressed.

DT Domain/ Component	Study/Population	Data/Clinical Assay	Analysis/Modelling Approaches	Validation Status DT Characteristics
A. Patient-Specific Computational Models and DT-like Approaches demonstrated in HNSCC				
Imaging	Aliotta et al. (2025) [9]: 72 patients, HPV-positive oropharyngeal carcinoma	Baseline: DW-MRI, FDG-PET and FMISO-PET Weekly intra-treatment during the first 3 weeks: MRI	Mechanistic radiobiological model	Single-centre retrospective study of prospectively collected trial data. No external validation. Patient-specific model capable of being longitudinally updated. However, it does not integrate molecular resistance data or simulation of alternative regimens. DT criteria: (i), (ii), (iii)
	Hormuth et al. (2026) [10]: 20 patients, HPV associated oropharyngeal carcinoma	Baseline and during radiotherapy: OE-MRI and DCE-MRI	Biology-based mathematical (coupled differential-equation) model	Small single-centre prospective clinical-trial cohort. Parameters calculated for 75% of the cohort and prediction in the held-out 25%. No external validation. Provided patient-specific imaging-based forecasting, but not longitudinal molecular integration. DT criteria: (i). Longitudinal imaging data was captured, but dynamic patient-specific model updating with new data was proposed as future perspectives.
Clinical Decision Support	Tardini et al. (2022) [12]: 536 patients with oropharyngeal squamous cell carcinoma	Clinical and demographic data, tumour staging; treatment history; radiomics from segmented primary tumours	Deep Reinforcement learning/Q-learning	Single-centre retrospective study. 75% of patients were used for training and 25% for held-out evaluation. It supports simulation of sequential treatment specific to each patient but does not demonstrate continuous updating with newly acquired longitudinal molecular data or prospective clinical utility. DT criteria: (i), (iv)
	Wentzel et al. (2024) [13]: 536 patients, oropharyngeal squamous cell carcinoma (same cohort as [12]).	Clinical, demographic, staging and radiotherapy-regimen data: age, sex, race, smoking status, nodal involvement, tumour stage and primary tumour site, radiation dose, treatment response, and dose-limiting toxicities.	Deep Reinforcement learning with visual explainability interface	No external or prospective clinical validation. Closest approximation to an operational HNSCC DT; however, it does not incorporate molecular signalling, ctDNA/exosomes or serial multimodal biological updates. DT criteria: (i), (iv), (ii) and (iii) are partially addressed.
Molecular/Multimodal Stratification	Islam et al. (2026) [104]: 1046 patients with HNC	Genomic, clinical and demographic data	Multimodal Machine Learning	Non-peer-reviewed preprint. This model allows for baseline multimodal stratification but does not incorporate longitudinal updating. DT criteria: (i)
B. DT-enabling Technologies relevant to Future Integration in HNSCC				
Imaging	Trada et al. (2023) [107]: 55 patients with non-metastatic mucosal HNSCC (37 oropharynx, 7 hypopharynx, 7 larynx, 4 nasopharynx)	Serial DWI-MRI (baseline, weeks 2,3,5,6 during RT; post-treatment) and FDG-PET/CT (baseline, week 3 and post-treatment)	Serial imaging analysis	Two prospective imaging biomarker studies. Imaging data were analysed as biomarkers rather than integrated into a continuously updated DT
Multi-omic Stratification	Wu et al. (2025) [102]: multi-omics cohort derived from oral cavity squamous cell carcinoma patients	Baseline characterisation: Genomic (WES, HPV genotyping), transcriptomic (RNAseq, qRT-PCR), proteomic and phosphoproteomic data (tandem mass tag), immunohistochemical analysis and treatment response experiments in OSCC patient-derived xenograft mouse models	Integrated multi-omic analysis and molecular stratification	Translation multi-cohort study. This study supports baseline stratification. Findings are specific to oral cavity squamous cell carcinoma, and its translation to HNSCC should be done carefully.
	Liu et al. (2025) [103]: 59,376 single cells and bulk RNA sequencing across ten datasets	Single-cell and bulk RNA sequencing	Molecular subtyping and machine-learning risk modelling	Multi-dataset analysis. Risk prediction was validated across four validation cohorts. Allows baseline stratification.
Liquid biopsy/resistance monitoring	Braig et al. (2016) [111]: 20 patients with HNSCC undergoing cetuximab-based therapy	Serial peripheral-blood ctDNA with targeted analysis of RAS and EGFR by next-generation sequencing	Serial ctDNA profiling to monitor RAS-mediated resistance	Clinical longitudinal molecular monitoring. Some mutations appeared during treatment and preceded clinical progression.
	Bruixola et al. (2025) [89]: 37 patients with locally advanced HNSCC treated with radical chemoradiotherapy.	Whole-exome sequencing of tumour tissue and ctDNA at diagnosis and relapse	Descriptive genomic profiling	Whole-exome ctDNA sequencing identified clonal changes and emerging-resistance-associated alterations. No such analysis was incorporated into a continuously updated DT.
Exosome-derived information	Ludwig et al. (2017) [121]: 38 HNC patients and 14 healthy donors	Collection of peripheral blood, followed by isolation of plasma-derived exosomes by mini-size-exclusion chromatography method; assessment of exosome morphology, size, number, and molecular/protein composition. Functional studies based on co-cultured assays with T and NK cell.	Exosome characterisation and functional immune-cell assays	Clinical biomarker study. Exosomal molecular content and immunosuppressive content were associated with disease activity. No integration into a continuously updated DT.
	Theodoraki et al. (2018) [123]: 40 HNSCC patients	Collection of peripheral blood, followed by isolation of plasma-derived exosomes by mini-size-exclusion chromatography method; detection of PD-L1 in exosomes by flow cytometry; clinicopathological correlation.	Exosome biomarker quantification and association analysis with tumour progression	Clinical biomarker study; not a prospective validation study. Exosome-derived molecular/immune information can be measured in plasma samples derived from HNSCC patients; longitudinal incorporation into continuously updating DTs remains unavailable.
Ex vivo Functional Testing	Millen et al. (2023) [131]: 97 patients, HNC biobank (110 models)	Patient-derived organoids; WES/NGS; RT, chemo- and targeted- agent screening	Establishment of a biobank of HNC organoids. Evaluation of the potential of these models as a tool to optimise therapeutic options.	Patient-derived organoids with predictive potential for therapeutic response and treatment strategy in HNC. Potential application of patient-specific organoids as input in DT, but not in longitudinal update.
PK/PD	Marin et al. (2026) [11]: 117 patients with recurrent or metastatic HNSCC treated with cetuximab	Serial plasma cetuximab concentrations, dosing records and clinical covariates (body surface area, age and serum albumin)	Population PK/PD modelling	Prospective, non-interventional, multicentre study. No independent external validation reported. This model is not a continuously updated patient-specific DT.
Toxicity Prediction	Chu et al. (2025) [143]: 1208 patients with HNC from two cohorts	Radiation-dose distribution, computed tomography imaging, organ-at-risk segmentations, baseline xerostomia score, age and sex	Three-Dimensional Deep Learning Model	In one cohort, 85% of patients were used for training and cross-validation, and the remaining 15% as an independent test set. The second cohort allowed for external validation of the model. Such a model could provide a toxicity-risk component within a future DT.
	Van den Bosch et al. (2021) [144]: 750 HNC patients	Patient electronic health records, ER, tumour characteristics, treatment regimen, radiation-induced toxicities at ten timepoints	Multivariable logistic prediction models	Prospective cohort study with external validation in an independent multicentre cohort (395 HNC patients). The integration of CITOR could add a multitoxicity risk assessment to the ideal DT for HNSCC.

## 7. Challenges and Clinical Translation

**Figure 2:**
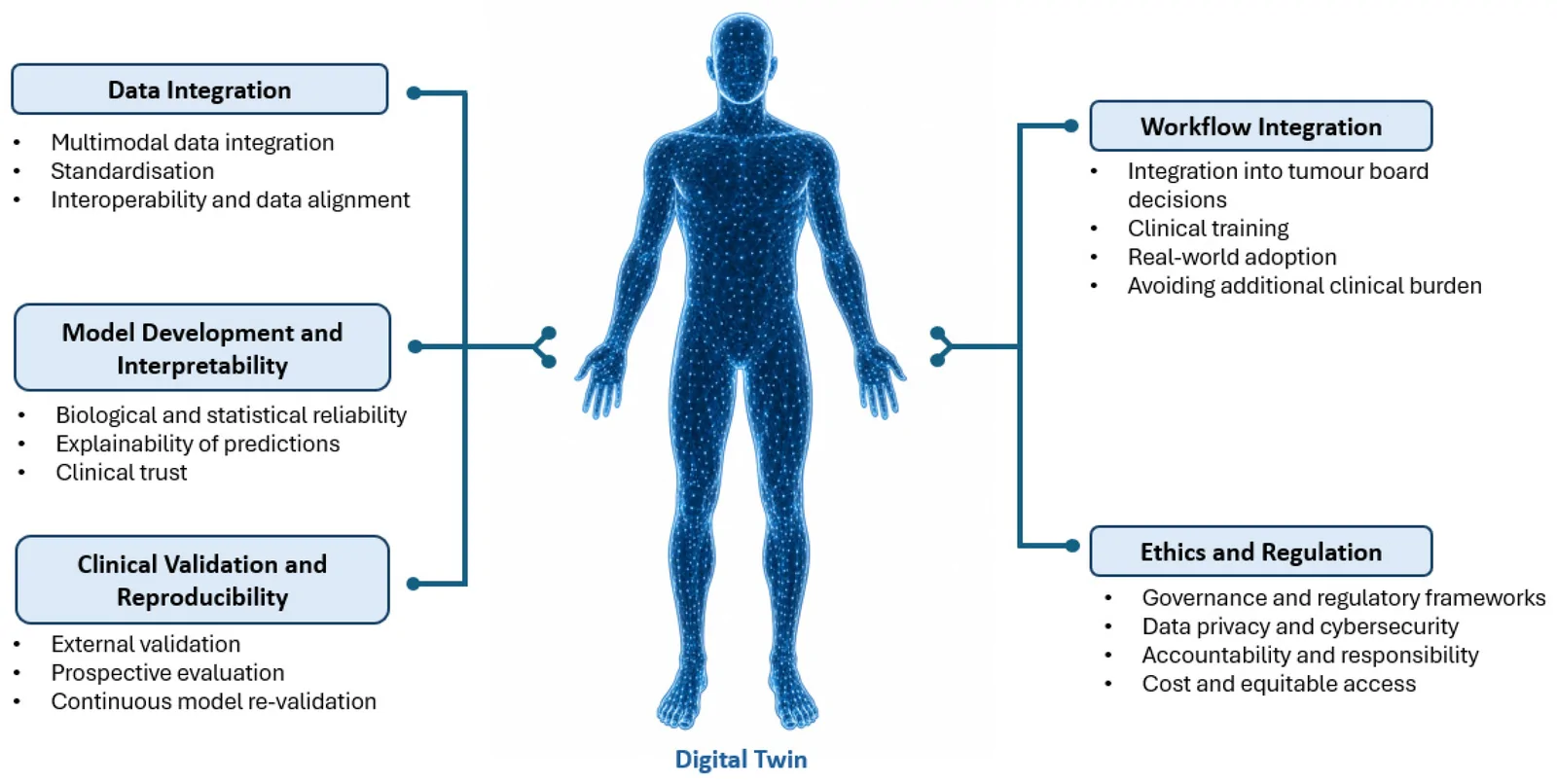
Principal challenges for the clinical translation of Digital Twins in HNSCC. The graphical element representing the Digital Twin (DT) was generated and refined with the assistance of the artificial intelligence (AI) tool ChatGPT, GPT-5.6 Sol (OpenAI).

The framework proposed in Section 6 remains for now a conceptual goal rather than a tool that has been put into clinical practice. Despite the promising potential of a DT for molecular stratification and the adaptation of therapeutic approaches in HNSCC, several challenges must be addressed (Figure 2).

### 7.1. Model Validation and Reproducibility

A key challenge to the clinical implementation of DTs is ensuring robust model validation and reproducibility, a process that requires extensive time, high-quality datasets, and substantial resources. Most of the models discussed in this review were developed and tested within single institutions. From the model described by Aliotta et al. [9] to the DITTO [13]/Tardini dyad [12], these have only been validated in a single cohort, with no external application of the model to date. At present, there is no standardised process for validating a DT, in the same way that pharmaceuticals or medical devices are validated [145]. The absence of such standards hampers the ability to ensure the fidelity and robustness of these new technologies [146]. This is particularly problematic for hybrid models that combine mechanistic and machine learning approaches, where there is a lack of protocols capable of validating the biological networks created or the statistical reliability [145]. Continuous re-validation is also important in an ideal HNSCC DT, as resistance mechanisms emerge during treatment, meaning a model’s predictions may deviate, even if its initial validation was robust.

A DT should also undergo a prospective clinical evaluation to confirm a real benefit to patient care compared with conventional clinical practices. In oncology, however, very few AI or DT tools are tested this way [147]. In practice, this is the stage at which most AI-based clinical decision support systems fail, as promising performance in in silico, retrospective or simulated data rarely translates into a demonstrated clinical benefit once tested in real patients. To address this gap, the Developmental and Exploratory Clinical Investigations of DEcision support systems driven by Artificial Intelligence (DECIDE-AI) guideline was recently created [148]. Standard randomised controlled trials used for medical validation also fit DTs poorly, due to their dynamic nature. Alternatives such as in silico trials and synthetic control arms must be taken into account in new trial designs [145], but no HNSCC-specific DT, including DITTO, has yet been evaluated this way.

In this way, the application of an HNSCC DT will need robust and standardised validation frameworks to ensure trust and to support clinical translation, including external multicentre validation, prospective evaluation of clinical utility, integration into routine workflows, and continuous updating as new patient-specific data become available.

### 7.2. Data Harmonisation, Standardisation, and Integration into Clinical Workflows

A validated model is still only as good as the data behind it, and this is where the next challenge arises. An ideal HNSCC DT would need to integrate multimodal data, ranging from genomic, imaging and liquid biopsy data to functional data obtained from different platforms. This in itself may already result in some technical noise. There is a lack of standard protocols and clinical interpretation guidelines, particularly for emerging biomarkers for HNSCC [50]. Imaging studies, laboratory systems and electronic health records also rarely share compatible formats or metadata, creating an additional challenge. Because of that, there is a need to transform these fragmented data sources into an interoperable data infrastructure [149,150]. This requires extensive preprocessing, including data cleaning, standardisation, normalisation and temporal alignment, to ensure trustworthy downstream modelling [145,150].

From an implementation perspective, adopting a DT may also present organisational challenges. Before its application, clinicians need training to understand how to interpret and act on the model’s outputs. Without this step, tools introduced without clinicians’ involvement tend to meet resistance, either because they are perceived as increasing workload or because staff were not involved in the tool’s development [145].

### 7.3. Interpretability and Trust in Models

In routine clinical practice, explanation is the core of any decision made for a patient. The clinician and patient need to understand each step of the diagnosis or treatment, in order to ensure transparency. The application of DTs should follow the same principle. The model must provide transparent and interpretable recommendations, allowing clinicians to understand the rationale underlying treatment decisions, such as the recommendation to discontinue cetuximab. Even if a model achieves high predictive performance, its recommendations are unlikely to be adopted if the reasoning behind them cannot be understood [151].

Among the models discussed in this review, DITTO is the only one that incorporates an explanatory interface, which its developers frame as a way of building clinical confidence [13].

### 7.4. Regulatory, Ethical, Legal, and Equity Considerations

Due to the recent emergence of the potential application of DT in healthcare, the applicable regulatory tools are still evolving. Regulatory agencies have already started to recognise these challenges through initiatives such as the Food and Drug Administration (FDA) Digital Health Center of Excellence and the European Medicines Agency (EMA) AI Action Plan [152]. Despite efforts, regulatory guidance remains fragmented, including long-term governance and implementation across different jurisdictions [145].

From an ethical perspective, DTs present numerous challenges that go beyond their technical complexity. Popa et al. (2021) [153] conducted a qualitative study combining a literature review with interviews involving partners from industry, academia, policy and civil society. This provided one of the first comprehensive assessments of the socio-ethical opportunities and risks associated with healthcare DTs. Data privacy stands out, as patient data may be shared with third parties or used against the patient’s interests, as well as being vulnerable to cybersecurity attacks if the data is not sufficiently protected. Another important question that remains unclear is who retains control of the data when the patient is treated at other institutions or even in other countries [145]. A separate question concerns legal responsibility and clinical authority: when a DT’s recommendation differs from a clinician’s judgement, whose view should prevail? In the review by Popa et al. (2021) [153], the majority of respondents agreed that DT could provide a range of clinical scenarios, but the final decision and the associated responsibility should remain with the clinician. This raises another equally important question: if a DT provides incorrect information or contributes to an inappropriate clinical decision, should accountability rest with the clinician, the developer, or the institution?

Cost and equitable access raise further concerns. Building and maintaining a dynamic system such as a DT requires a multidisciplinary team, significant computational power, as well as specialised personnel to operate it, resulting in high costs. Consequently, not all medical centres worldwide will have easy access to these tools, which risks limiting DT-based care to well-resourced institutions [145]. The potential monopolisation of DT technology raises a special concern if these platforms become patented and commercialised [154]. If there are no agreements on equitable access, the high costs could result in widening healthcare disparities.

Additionally, the actual implementation of the model must be designed to address human diversity in its entirety (genetic, socio-economic, environmental) and not be limited to specific population groups within society, to enable equitable clinical translation [153].

## 8. Conclusions and Future Perspectives

This review demonstrated that no currently available model integrates the molecular and pharmacodynamic dimensions of HNSCC (genomic and multi-omic stratification, predictive resistance biomarkers, functional drug-response data) with the clinical decision counterpart. We describe how different data modalities could contribute complementary information to our conceptual DT framework. Longitudinal imaging data could provide the basis for the DT by representing tumour dynamics through biologically based mathematical models. The inclusion of a multi-omics data layer allows not only the molecular stratification of patients but also the identification of molecular changes that should be monitored longitudinally. Through liquid biopsies collected between treatment cycles, we can monitor these changes and potentially predict emerging resistance mechanisms. If detected, the DT should be able to assist the evaluation of alternative regimens suitable for the patient. Through PK/PD models, we can also understand the patient’s dose-response relationship, as well as drug distribution, whereas functional studies could provide complementary information on tumour sensitivity to different therapies or drug combinations. This approach is achieved through a hybrid architecture, which allows each type of data to be handled by the most appropriate computing system.

Among the reviewed frameworks, the deep reinforcement learning framework underpinning DITTO comes closest to an ideal DT, although it was developed specifically for oropharyngeal squamous cell carcinoma. This tool predicts survival, toxicity and treatment decisions with meaningful accuracy. Nevertheless, it does not integrate the molecular signalling pathways (EGFR, PIK3/AKT/mTOR/HRAS), longitudinal evaluation of predictive resistance biomarkers, or even imaging data. This gap between molecular resolution and clinical practice is, in our view, the central obstacle to translating the DT concept into prediction for targeted therapy in HNSCC.

The DT framework proposed in this review aims to assist clinicians in selecting the most appropriate therapeutic strategies for each HNSCC patient. Hence, the DT must combine imaging, genomic/multi-omic stratification, longitudinal liquid biopsy, and ex vivo functional testing into a single, continuously updated model.

Future work should focus on generating longitudinal datasets that integrate and harmonise clinical, molecular, imaging and functional information, as well as on developing mathematical and computational models capable of representing the dynamic progression of HNSCC. Finally, their validation will be the key to their future application, enabling DTs to become clinical support tools, playing an essential role in facilitating clinical decision-making that is best suited to HNSCC patients. In the future, this DT architecture may contribute to a shift in HNSCC management from static, pre-treatment biomarker selection toward an adaptive and dynamic framework capable of anticipating resistance and supporting personalised treatment decisions.

## Data Availability

The original contributions presented in this study are included in the article. Further inquiries can be directed to the corresponding author.

## Abbreviations

The following abbreviations are used in this manuscript:

DT	Digital Twin
HNSCC	Head and Neck Squamous Cell Carcinoma
ctDNA	circulating tumour DNA
PK/PD	Pharmacokinetic/Pharmacodynamic
EBV	Epstein-Barr Virus
HPV	Human Papillomavirus
HNC	Head and Neck Cancer
USA	United States of America
MATH	Mutant-Allele Tumour Heterogeneity
EGFR	Epidermal Growth Factor Receptor
HER3	Human Epidermal Growth Factor Receptor 3
STAT3	Signal Transducer and Activator of Transcription 3
TP53	Tumour Protein 53
CDKN2A	Cyclin-Dependent Kinase Inhibitor 2A
PIK3CA	Phosphatidylinositol-4,5-Bisphosphate 3-Kinase Catalytic Subunit Alpha
NOTCH1	Notch Receptor 1
PD-L1	Programmed Death-Ligand 1
HRAS	Harvey Rat Sarcoma Viral Oncogene Homolog
RTKs	Receptor Tyrosine Kinases
PI3K	Phosphoinositide 3-Kinase
AKT	Protein Kinase B
AXL	AXL Receptor Tyrosine Kinase
mTOR	Mechanistic Target of Rapamycin
PTEN	Phosphatase and Tensin Homolog
CCND1	Cyclin D1
KRAS	Kirsten Rat Sarcoma Viral Oncogene Homolog
NRAS	Neuroblastoma RAS Viral Oncogene Homolog
NRG1	Neuregulin 1
RAF	Rapidly Accelerated Fibrosarcoma
MEK	Mitogen-Activated Protein Kinase Kinase
ERK	Extracellular Signal-Regulated Kinase
MAPK	Mitogen-Activated Protein Kinase
TCGA	The Cancer Genome Atlas
SOX2	SRY-Box Transcription Factor 2
CD56	Neural Cell Adhesion Molecule
CAF	Cancer-associated Fibroblasts
HLA	Human Leucocyte Antigen
VEGF	Vascular Endothelial Growth Factor
IL-6	Interleukin-6
CPS	Combined Positive Score
ERBB2	Erb-B2 Receptor Tyrosine Kinase 2
HER2	Human Epidermal Growth Factor Receptor 2
MET	Mesenchymal-Epithelial Transition Factor
miRNA	microRNA
IGF-1R	Insulin-like Growth Factor 1 Receptor
HGF	Hepatocyte Growth Factor
HIF	Hypoxia-inducible Factor
EMT	Epithelial-to-Mesenchymal Transition
EMT-TFs	Epithelial-to-Mesenchymal Transition Transcription Factors
PPP	Picropodophyllin
CRISPR/Cas9	Clustered Regularly Interspaced Short Palindromic Repeats/CRISPR-associated protein 9
EGF	Epidermal Growth Factor
CSC	Cancer Stem Cell
WES	Whole-Exome Sequencing
ICI	Immune Checkpoint Inhibitors
IFN-γ	Interferon-gamma
LAG-3	Lymphocyte-Activation Gene 3
TIM-3	T-cell Immunoglobulin and Mucin-domain containing-3
VISTA	V-domain Ig Suppressor of T-cell Activation
NASA	National Aeronautics and Space Administration
AI	Artificial Intelligence
DW-MRI	Diffusion-Weighted Magnetic Resonance Imaging
PET	Positron Emission Tomography
FDG	Fluorodeoxyglucose
FMISO	Fluoromisonidazole
FGF	Fibroblast Growth Factor
DWI	Diffusion-Weighted Imaging
MRI	Magnetic Resonance Imaging
CT	Computed Tomography
OSCC	Oral Cavity Squamous Cell Carcinoma
PDX	Patient-Derived Xenograft
RRAS	Related RAS viral oncogene homolog
cfDNA	Cell-free DNA
ddPCR	Droplet digital PCR
BEAMing	Beads, Emulsion, Amplification and Magnetics
PK	Pharmacokinetics
ALI	Air-Liquid Interface
NTCP	Normal Tissue Complication Probability
CITOR	Comprehensive Individual Toxicity Risk Profile
XAI	Explainable Artificial Intelligence
ORIEN	Oncology Research Information Exchange Network
FDA	Food and Drug Administration
EMA	European Medicines Agency
